# Effects of Omega-3 Fatty Acids Supplementation on Serum Lipid Profile and Blood Pressure in Patients with Metabolic Syndrome: A Systematic Review and Meta-Analysis of Randomized Controlled Trials

**DOI:** 10.3390/foods12040725

**Published:** 2023-02-07

**Authors:** Yin-Xiu Liu, Jun-Hui Yu, Ji-Han Sun, Wen-Qin Ma, Jin-Jing Wang, Gui-Ju Sun

**Affiliations:** 1 Key Laboratory of Environmental Medicine and Engineering of Ministry of Education, School of Public Health, Southeast University, Nanjing 210009, China; 2 Department of Nutrition and Food Hygiene, School of Public Health, Southeast University, Nanjing 210009, China; 3 Rugao People’s Hospital, Nantong 226000, China

**Keywords:** omega-3 polyunsaturated fatty acids, metabolic syndrome, blood lipid, blood pressure, meta-analysis

## Abstract

The purpose of this study was to explore the effect of omega-3 polyunsaturated fatty acids (n-3 PUFAs) supplementation on serum lipid profile and blood pressure in patients with metabolic syndrome. We searched PubMed, Web of Science, Embase, and the Cochrane library from database inception to 30 April 2022. This meta-analysis included eight trials with 387 participants. We found that supplementation of n-3 PUFAs has no significant reduction in TC level (SMD = −0.02; 95% CI: −0.22 ~ 0.18, I^2^ = 23.7%) and LDL-c level in serum (SMD = 0.18; 95% CI: −0.18 ~ 0.53, I^2^ = 54.9%) of patients with metabolic syndrome. Moreover, we found no significant increase in serum high-density lipoprotein cholesterol level (SMD = 0.02; 95% CI: −0.21 ~ 0.25, I^2^ = 0%) in patients with metabolic syndrome after consuming n-3 PUFAs. In addition, we found that n-3 PUFAs can significantly decrease serum triglyceride levels (SMD= −0.39; 95% CI: −0.59 ~ −0.18, I^2^ = 17.2%), systolic blood pressure (SMD = −0.54; 95% CI: −0.86 ~ −0.22, I^2^ = 48.6%), and diastolic blood pressure (SMD = −0.56; 95% CI: −0.79 ~ 0.33, I^2^ = 14.0%) in patients with metabolic syndrome. The results from the sensitivity analysis confirmed that our results were robust. These findings suggest that n-3 PUFA supplementation may serve as a potential dietary supplement for improving lipids and blood pressure in metabolic syndrome. Given the quality of the included studies, further studies are still needed to verify our findings.

## 1. Introduction

Metabolic syndrome (MetS) is a group of disorders that cause disturbed metabolism, including abdominal obesity, insulin resistance, hypertension, and dyslipidemia [1,2]. Metabolic syndrome, often associated with an elevated risk of cardiovascular disease [3,4], has become a major health problem and affects approximately one-quarter of North Americans [1,5]. People with MetS may have a higher risk of coronary heart disease and stroke than those without MetS [5,6]. Thus, prevention and treatment of metabolic syndrome will greatly benefit from the exploration of protective factors.

Studies have found that dietary patterns are associated with metabolic syndrome [7,8,9]. In the human diet, dietary lipids play an important role in affecting health and physiological functions [10,11,12]. There is evidence that unsaturated fatty acids are more beneficial to health than saturated fatty acids [11,13]. Omega-3 polyunsaturated fatty acids (n-3 PUFAs) can be divided into animal and plant sources, with plant sources such as linolenic acid and animal sources such as Docosahexaenoic Acid (DHA) and Eicosatetraenoic Acid (EPA) from fish. n-3 PUFAs have cardioprotective, anti-inflammatory, and triglyceride-lowering properties, so they may help treat obesity and improve metabolic syndrome [14,15,16].

However, few studies have explored the effects of n-3 PUFAs on serum lipid profile and blood pressure in patients with metabolic syndrome. A recent meta-analysis exploring the effects of dietary EPA and DHA supplementation on metabolic syndrome showed that both EPA and DHA were effective in reducing serum triglyceride (TG) levels and that EPA and DHA had different effects on risk factors for MetS [17]. In addition, several studies have focused on the relationship between n-3 PUFAs and the risk of metabolic syndrome [18,19]. The efficacy of n-3 PUFAs on metabolic syndrome, especially in humans, remains controversial [20]. Therefore, it is essential to study the effect of n-3 PUFAs on serum lipid profile and blood pressure in people with metabolic syndrome. This study intended to explore the effects of n-3 PUFAs on lipid profile and blood pressure in patients with MetS.

## 2. Materials and Methods

We prospectively registered the study protocol at PROSPERO (https://www.crd.york.ac.uk/prospero/, accessed on 1 December 2022) as identifier CRD42022372477. We reported this systematic review and meta-analysis following the preferred 2020 reporting items for systematic reviews and meta-analyses (PRISMA) guidelines [21].

### 2.1. Literature Search Strategy

The PubMed database, Web of Science database, Embase database, and Cochrane Library database were systematically searched for relevant articles up to 30 April 2022, using subject words combined with the free words method. Only English-language publications were included. Additionally, the references list of the relevant articles was retrieved. The detailed search strategy is outlined in Appendix A.

### 2.2. Inclusion and Exclusion Criteria

The trials were included if they met the following criteria: (a) the type of study design is limited to randomized controlled trials (RCTs), which could either be of parallel group or crossover design; (b) adult participants aged or more than 18 years old diagnosed with metabolic syndrome; (c) participants in the intervention group take omega-3 fatty acids or foods enriched in omega-3 fatty acids; and (d) the outcomes included total cholesterol (TC), triglyceride (TG), high-density lipoprotein cholesterol (HDL-c), low-density lipoprotein cholesterol (LDL-c), systolic blood pressure (SBP), and diastolic blood pressure (DBP). Studies that include either of these outcomes were considered for inclusion. Exclusion criteria were listed as follows: (a) randomized trials conducted in special populations such as pregnant women; (b) trials that lack a control group; (c) the intervention included other supplements of no interest; and (d) animal or cell experiments, case reports, comments, letters, editorials, conference papers, and literature with unavailable or unconverted data.

### 2.3. Data Extraction and Methodological Assessment

Two reviewers extracted relevant information independently using spreadsheets. We extracted the following information from eligible studies: name of the first author, year of publication, study country, sample size, dose and type of the intervention, control group, study duration, and outcomes. We would have contacted relevant authors for raw data if outcome data were missing. Standard deviation and mean were calculated using appropriate methods supported by the relevant literature when available [22,23,24].

We assessed the quality of the studies using the Cochrane Collaborations tool. The assessment consisted of seven items: random sequence generation (selection bias); allocation concealment (selection bias); blinding of participants and personnel (performance bias); blinding of outcome assessment (detection bias); incomplete outcome data (attrition bias); and selective reporting (reporting bias) and other biases. Each item can be graded as low risk, high risk, or unclear risk (if there is insufficient information).

### 2.4. Data Synthesis and Analysis

Stata SE 17.0 (Stata Corporation, TX, USA) was used to conduct the statistical analyses. When there were multiple endpoints in the trial, we used the last end value. In each study, we presented estimates using standardized mean difference (SMD) with 95% confidence intervals. The significance level was two-sided, *p* < 0.05. The I^2^ metrics and chi-square statistics were used to assess the heterogeneity. The random-effect model or the fixed-effect model was selected according to the degree of heterogeneity. Additionally, a sensitivity analysis was performed to appraise the stability of our results. Subgroup analyses were used to determine potential influencing factors, including study design, study quality, the type of control, and intervention duration. Egger’s test was conducted if there were more than 10 studies, and the funnel plot was used to detect potential publication bias.

## 3. Results

### 3.1. Description of Selected Trials

**Figure 1:**
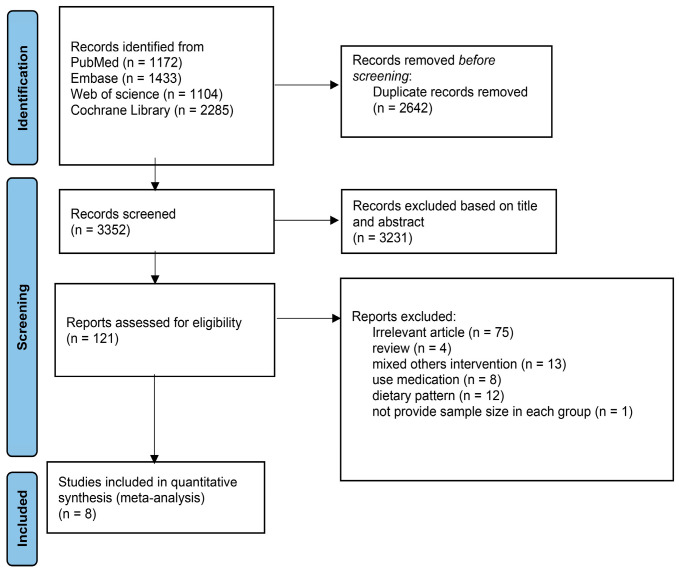
Study selection process based on PRISMA guideline.

According to the search strategy, 5994 articles from the database were retrieved. The remaining records were 3352 after all duplicates were removed. Based on the titles and abstracts, 3231 studies were excluded, including animal experiments, non-RCTs, literature reviews, or studies that were not relevant to n-3 PUFAs. After screening the full text of the remaining 121 articles, 101 articles were excluded. The reasons were as follows: (i) irrelevant article (n = 75); (ii) review (n = 4); (iii) mixed others intervention (n = 13); (iv) use medication (n = 8); (v) dietary pattern (n = 12); (vi) did not provide sample size in each group (n = 1). Finally, eight trials with enough data were selected in this meta-analysis. The flow chart of selection is shown in Figure 1.

### 3.2. Study Characteristics

The basic characteristics of included trials are presented in Table 1. In total, 387 adults with metabolic syndrome were enrolled. The publication year of included trials was between 2009 and 2019. Study duration ranged from 49 days to 168 days. One study was a crossover trial, whereas the remaining seven studies were parallel-controlled trials. The mean age of patients from included trials was 45.54 years old.

### 3.3. Risk of Bias Assessment

**Figure 2:**
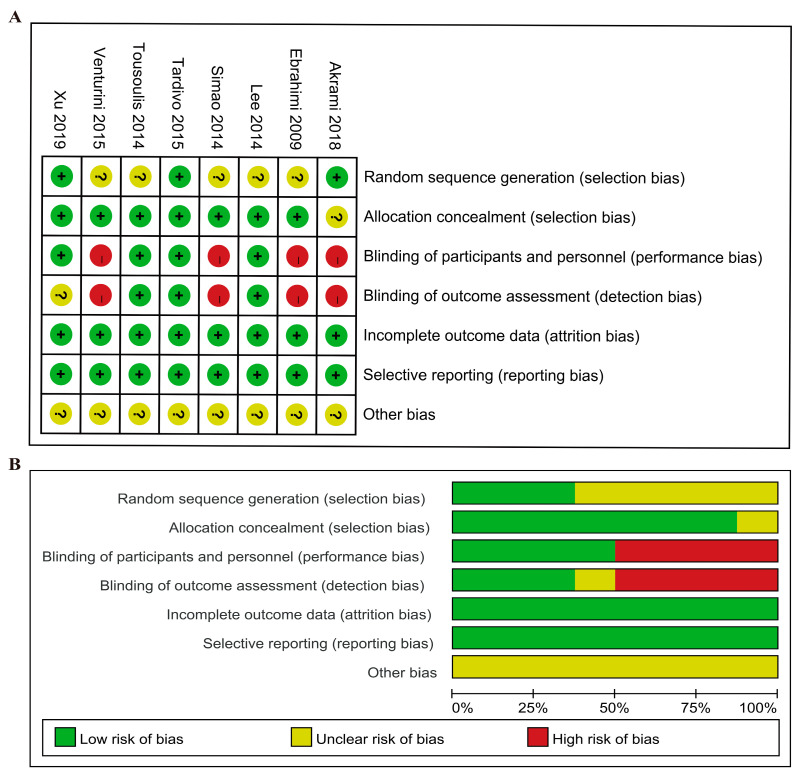
The assessment of bias risk of included studies: (**A**) Bias risk summary. Bias risk was classified as low (+), unclear (?), or high (−). (**B**) Bias risk graph. Reviewing authors’ judgments about the bias risk of each item, they were shown as percentages across all included studies.

In terms of risk of bias, five trials were categorized as with an unclear risk of bias, and three trials were classified as having a low risk of bias in random sequence generation. In the domain of allocation concealment, seven trials were rated low risk, and one trial with unclear risk of bias. Four trials were evaluated as with high risk in the domain of blinding of participants, personnel, and outcome assessment. Eight trials were rated low risk in the domain of incomplete outcome data and in the domain of selective reporting. Overall, four trials were rated low risk, and four were high risk (Figure 2).

### 3.4. Low-Density Lipoprotein Cholesterol

**Figure 3:**
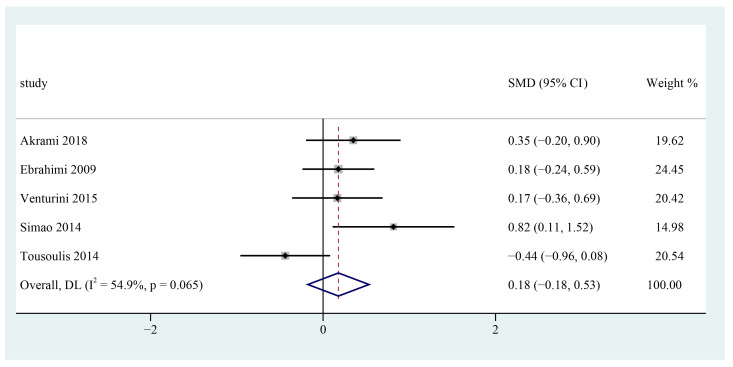
Effect of omega-3 fatty acids supplementation on LDL-C (mmol/L) in patients with metabolic syndrome. Open diamonds: synthesis of summary results of all studies; Filled diamonds: point estimates for effects of each study; Dashed lines: summary effect estimate which is labeled as a dashed line perpendicular to the X axis; Grey squares: the weight of each study, the value of weigh is proportional to the diamond size; SMD: standardized mean difference.

Five studies investigated the effects of supplementing n-3 PUFAs on serum LDL-c in patients with metabolic syndrome [25,26,27,28,29]. Based on a meta-analysis of data from the included trials, it was indicated that supplementing n-3 PUFAs has no significant reduction in serum LDL-c level among patients with metabolic syndrome (SMD = 0.18; 95% CI: −0.18 ~ 0.53, I^2^ = 54.9%) (Figure 3).

### 3.5. High-Density Lipoprotein Cholesterol

**Figure 4:**
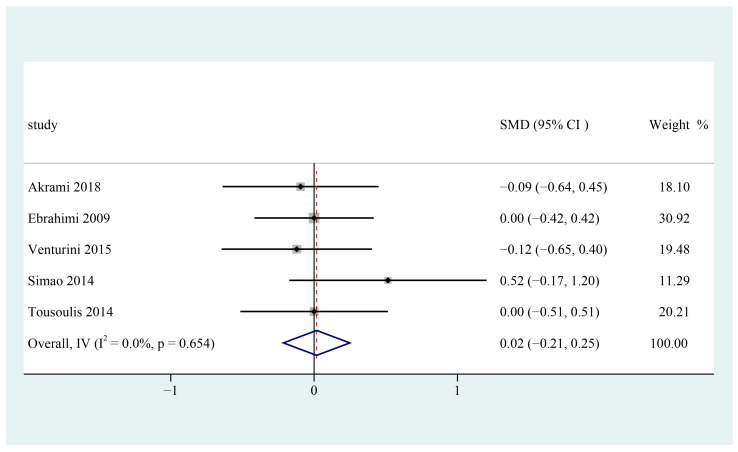
Effect of omega-3 fatty acids supplementation on HDL-C (mmol/L) in patients with metabolic syndrome. Open diamonds: synthesis of summary results of all studies; Filled diamonds: point estimates for effects of each study; Dashed lines: summary effect estimate which is labeled as a dashed line perpendicular to the X axis; Grey squares: the weight of each study, the value of weigh is proportional to the diamond size; SMD: standardized mean difference.

Five studies explored n-3 PUFA supplements’ effects on serum HDL-c in patients with metabolic syndrome [25,26,27,28,29]. The pooled result revealed that supplementing n-3 PUFAs did not significantly increase serum high-density lipoprotein cholesterol levels among patients with metabolic syndrome (SMD = 0.02; 95% CI: −0.21 ~ 0.25, I^2^ = 0%) (Figure 4).

### 3.6. Total Cholesterol

**Figure 5:**
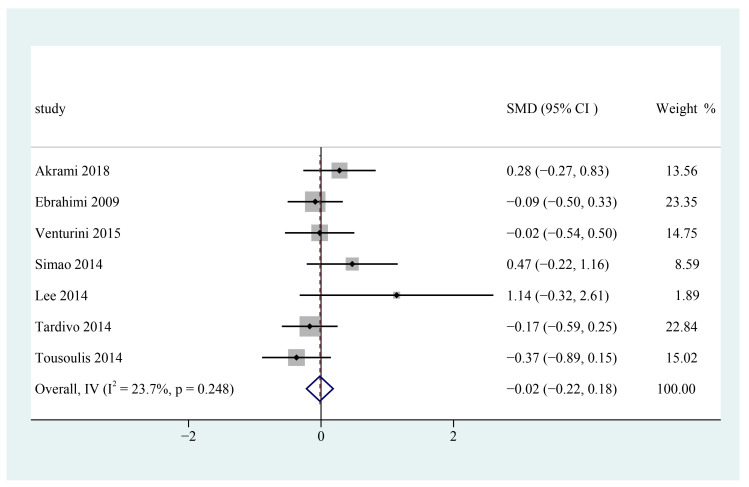
Effect of omega-3 fatty acids supplementation on TC (mmol/L) in patients with metabolic syndrome. Open diamonds: synthesis of summary results of all studies; Filled diamonds: point estimates for effects of each study; Dashed lines: summary effect estimate which is labeled as a dashed line perpendicular to the X axis; Grey squares: the weight of each study, the value of weigh is proportional to the diamond size; SMD: standardized mean difference.

Seven trials appraised the effects of n-3 PUFAs on serum TC in patients with metabolic syndrome [25,26,27,28,29,30,31]. The pooled results indicated that n-3 PUFA consumption has no significant reduction in serum total cholesterol level in patients with metabolic syndrome (SMD = −0.02; 95% CI: −0.22~0.18, I^2^ = 23.7%) (Figure 5).

### 3.7. Triglycerides

**Figure 6:**
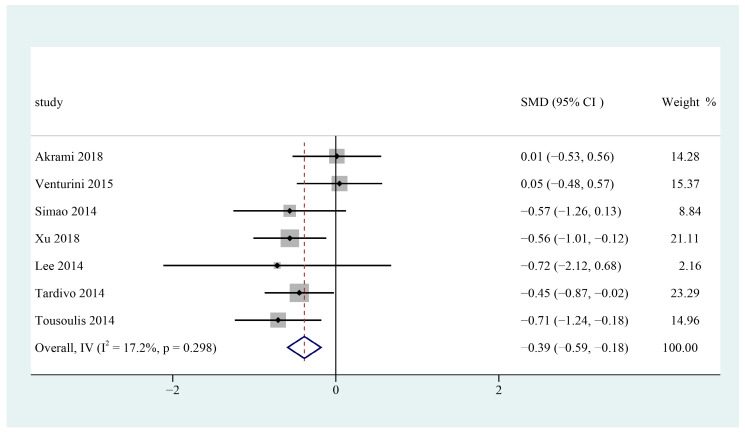
Effect of omega-3 fatty acids supplementation on TG (mmol/L) in patients with metabolic syndrome. Open diamonds: synthesis of summary results of all studies; Filled diamonds: point estimates for effects of each study; Dashed lines: summary effect estimate which is labeled as a dashed line perpendicular to the X axis; Grey squares: the weight of each study, the value of weigh is proportional to the diamond size; SMD: standardized mean difference.

Seven trials were included to pool the data for the meta-analysis [25,27,28,29,30,31,32]. The results showed that consuming n-3 PUFAs supplement may significantly decrease serum triglyceride levels in patients with metabolic syndrome (SMD = −0.39; 95% CI: −0.59 ~ −0.18, I^2^ = 17.2%) (Figure 6).

### 3.8. Systolic Blood Pressure

**Figure 7:**
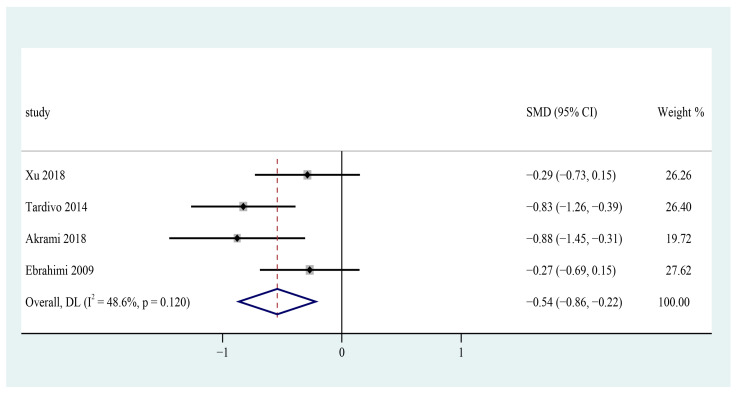
Effect of omega-3 fatty acids supplementation on SBP (mmHg) in patients with metabolic syndrome. Open diamonds: synthesis of summary results of all studies; Filled diamonds: point estimates for effects of each study; Dashed lines: summary effect estimate which is labeled as a dashed line perpendicular to the X axis; Grey squares: the weight of each study, the value of weigh is proportional to the diamond size; SMD: standardized mean difference.

Four trials were included to pool the data for the meta-analysis [25,26,31,32]. The results showed that consuming n-3 PUFAs supplement may significantly decrease systolic blood pressure in patients with metabolic syndrome (SMD = −0.54; 95% CI: −0.86 ~ −0.22, I^2^ = 48.6%) (Figure 7).

### 3.9. Diastolic Blood Pressure

**Figure 8:**
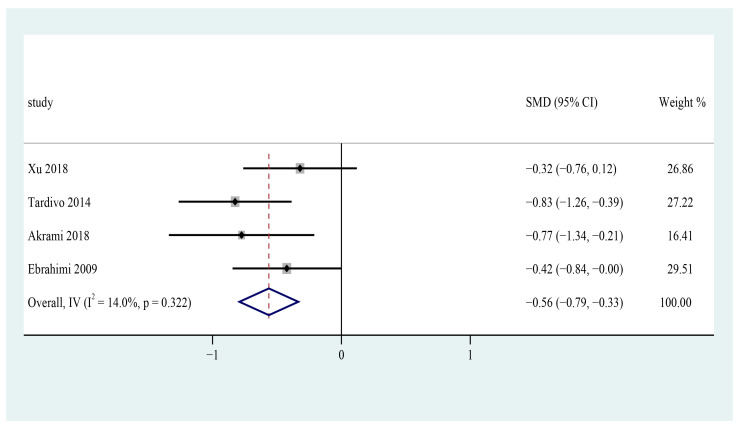
Effect of omega-3 fatty acids supplementation on DBP (mmHg) in patients with metabolic syndrome. Open diamonds: synthesis of summary results of all studies; Filled diamonds: point estimates for effects of each study; Dashed lines: summary effect estimate which is labeled as a dashed line perpendicular to the X axis; Grey squares: the weight of each study, the value of weigh is proportional to the diamond size; SMD: standardized mean difference.

Four trials appraised the effects of n-3 PUFAs on diastolic blood pressure in patients with metabolic syndrome [25,26,31,32]. The pooled results revealed that n-3 PUFA consumption could significantly reduce diastolic blood pressure in patients with metabolic syndrome (SMD = −0.56; 95% CI: −0.79~ −0.33, I^2^ = 14.0%) (Figure 8).

### 3.10. Subgroup Analysis and Sensitivity Analysis

Table S1 presents the results of the subgroup analysis. Systolic and diastolic blood pressures were not analyzed by subgroups due to the limited number of studies. For serum TC, LDL-c, and HDL-c, we did not observe significant differences in effects by subgroup in studies. Moreover, we found that a significant decrease in TG concentration in serum was seen in a study lasting more than 12 weeks. The results of the sensitivity analysis are presented in Figures S1–S6. By using sensitivity analysis, we confirmed that our results are reliable and robust.

### 3.11. Publication Bias

**Figure 9:**
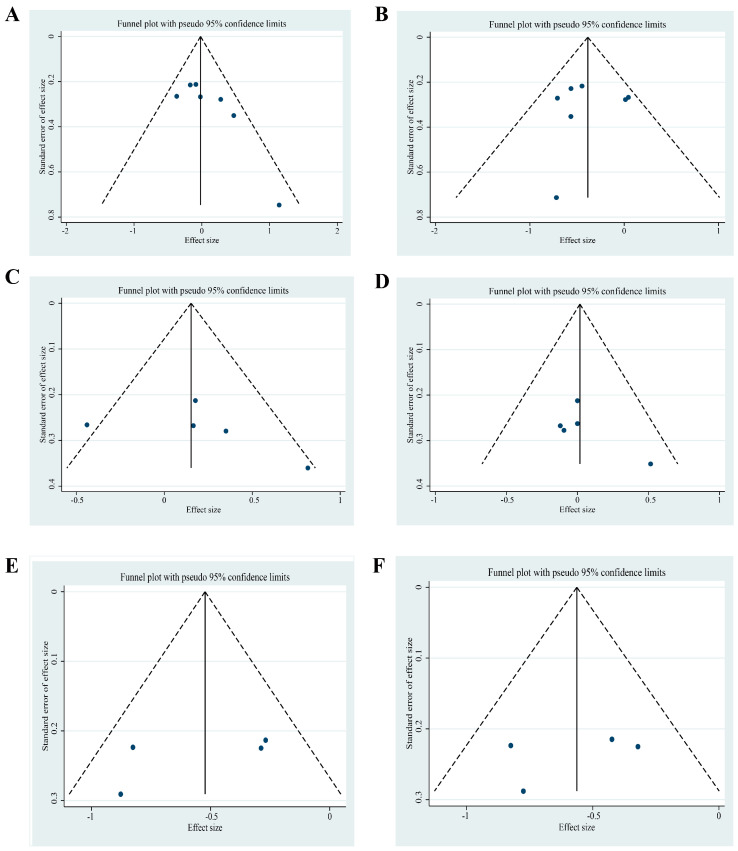
Funnel plots to evaluate publication bias and effect of omega-3 fatty acids supplementation for (**A**) TC, (**B**) TG, (**C**) LDL-c, (**D**) HDL-c, (**E**) SBP, and (**F**) DBP in patients with metabolic syndrome.

Funnel plots were used to evaluate publication bias. Based on visual scanning of the funnel plot, it was evident there was slight asymmetry due to the limited number of studies among all outcomes (Figure 9).

## 4. Discussion

In this study, we reviewed the literature and performed a meta-analysis to examine the effects of n-3 PUFAs consumption on serum lipid profile and blood pressure among patients with metabolic syndrome. We found no significant changes in serum TC, LDL-c, and HDL-c following supplementation with n-3 PUFAs in patients with metabolic syndrome. Additionally, we found a significant reduction in serum TG, SBP, and DBP following consuming n-3 PUFAs in patients with metabolic syndrome. The pooled estimates were relatively robust for all outcomes.

Omega 3 PUFAs have been linked to improvement in lipid profile [14,33,34]. Our meta-analysis included relevant randomized controlled trials and confirmed that n-3 PUFAs could reduce serum TG levels. A sufficient intake of n-3 PUFAs can prevent hypertriglyceridemia by inhibiting the liver’s ability to produce triglycerides and low-density lipoproteins [35]. In vitro, peroxisome proliferator-activated receptors can be stimulated by polyunsaturated fatty acids, leading to adipocyte differentiation and accelerated maturation [36,37]. Adipogenesis and healthy expansion of adipose tissue are promoted by n-3 PUFAs in the presence of a positive energy balance, thus contributing to a healthy metabolic phenotype.

A recent study by Mates et al. [38] concluded that walnut-enriched diets decreased triglyceride, total cholesterol, and LDL cholesterol concentrations. Walnuts are important plant sources of omega-3 PUFAs, which are especially rich in linoleic acid, α-linolenic acid, polyphenols, and magnesium. Our results indicated that consuming n-3 PUFAs may cause a significant reduction in serum TG among patients with metabolic syndrome. We did not find that n-3 PUFAs could lower total cholesterol and LDL cholesterol. In our analysis, we consider that the beneficial effects of a diet rich in walnuts may be due to the combined effects of the multiple nutrients in walnuts, not just the effects of α-linolenic acid alone. Our current study focuses solely on the effects of n-3 PUFAs alone, as the effects may not be superior to the effects of interventions with a walnut-rich diet. In addition, the inclusion criteria of the study by Mates et al. [38] did not coincide with the inclusion criteria of our study. Therefore, there was some variability in the final characteristics of participants, which could lead to results that are not entirely consistent between the two studies.

Our results indicated that consuming n-3 PUFAs may benefit patients with metabolic syndrome by improving lipid profiles. A meta-analysis in 2017 revealed that high blood levels of n-3 PUFAs were associated with a lower risk of metabolic syndrome [39]. The following mechanisms may explain why FUFAs reduce the risk of metabolic syndrome. First, adequate intake of n-3 PUFAs may reduce triglyceride and LDL synthesis in the liver. In addition, n-3 PUFAs lower blood pressure by reducing angiotensin-converting enzyme levels in the aorta. Finally, n-3 PUFAs can increase insulin sensitivity and prevent hyperglycemia [40,41].

Adipogenic genes are inhibited by n-3 PUFAs. Mitochondrial and peroxisomal fatty acid oxidation genes are activated by n-3 PUFAs. n-3 PUFAs have cardiovascular protective effects and improve endothelial function [42]. The type of n-3 PUFAs may have different roles in regulating lipid metabolism [43]. We did not observe a reduction in serum LDL-c level or an increase in serum HDL-c level. Our current included trials did not specifically break out the different types of n-3 PUFAs due to the limited number of studies but rather explored the effects on lipid profiles from an overall perspective, and thus our results may be biased by differences in the effects of different types of unsaturated fatty acids that may lead to a final overall effect. Thus, this may explain the contradiction between the effects of some endings and our assumptions. n-3 PUFAs reduce adipogenesis and low-density lipoprotein production in the liver. As a result, n-3 PUFAs limit the supply of fatty acids to fat cells, thereby reducing triglyceride levels [44]. Overall, n-3 PUFAs regulate lipid metabolism, facilitate fatty acid oxidation, inhibit lipogenesis, and promote lipid distribution and adipocyte metabolism.

A meta-analysis from 33 randomized controlled trials conducted by Zhang et al. [17] concluded that both EPA and DHA were effective at lowering serum TG levels. EPA supplementation decreased TC, TG, and LDL-C, while DHA increased the serum levels of TC, LDL-C, and HDL-C. Moreover, DHA increased the serum levels of insulin compared with EPA, especially in subgroups whose mean age was <60 years and whose duration of DHA supplementation <3 months. This study aimed to clarify whether EPA and DHA have differential effects on MetS features in humans. Unlike the purpose of this study, we focused on exploring the effects of n-3 PUFAs on lipid profiles and blood pressure in people diagnosed with metabolic syndrome. There are several reasons why we have a low number of eligible literature. Firstly, there is a discrepancy between the search database of our current study and Zhang’s study. Secondly, of the 33 studies included in Zhang’s study [17], 22 trials included at least one chronic disease, and 10 trials included healthy subjects. This is completely different from the inclusion criteria of our current study. Thirdly, in terms of search terms, Zhang’s study searched for DHA and EPA as keywords (with requirements also for ratio and purity), whereas our study did not restrict these elements. Thus, there will be relatively less literature that meets the requirements of our study.

Hypertension is characterized by an imbalance between vasoconstriction and vasodilation. It is influenced by genetic variation and environmental risk factors such as an unhealthy diet. Numerous interventional clinical trials and cross-sectional analyses have shown that supplementation with n-3 PUFAs can reduce blood pressure in both hypertensive and normal populations [45]. This is consistent with the trend of results found in our current study. The protective mechanisms in preventing hypertension for n-3 PUFAs may be as follows: n-3 PUFAs/n-3 oxylipins can reduce oxidative/nitrative stress and regulate the function of the membrane by influencing ion channels and receptors and altering the structure and function of the cell surface microdomain. Furthermore, the protective effects of n-3 PUFAs/n-3 oxylipins on hypertension involve the metabolic and functional competition with n-6 PUFAs/n-6 oxylipins [46].

In the current study, we only included eight trials in the meta-analysis. There were seven trials involving the use of DHA/EPA supplements and one trial involving flaxseed oil (linolenic acid C81:3 omega 3 supplements). Flaxseed and flaxseed oil are rich sources of alpha-linolenic acid (ALA). n-3 PUFAs are found in food in three forms: ALA, EPA, and DHA. ALA is mainly found in plant foods, such as vegetables, fruits, nuts, soybeans, and vegetable oils. EPA mainly comes from marine animals, such as fish, and shrimp, especially deep-sea fish. Like EPA, the main sources of DHA are fish, shrimp, and deep-sea fish [47]. It should be noted that ALA, EPA, and DHA are three types of n-3 PUFAs [48,49], which are different from each other in terms of their effects on the human body. ALA helps prevent the formation of cholesterol deposits in blood vessels, reduces inflammation in arteries, and inhibits tumor growth. EPA is mainly anti-inflammatory and has a relieving effect on cardiovascular disease and arthritis. DHA is for nerve regeneration and helps improve cognitive function, memory, and concentration [49]. Another difference between ALA and DHA and EPA is that ALA must be converted into DHA and EPA in the body to be used by the body, but the conversion speed is slower; the conversion rate is not high; and eating vegetables, vegetable oils, or flax cannot produce enough EPA and DHA for the body. Our current study explored the overall effect of n-3 PUFAs supplementation, and due to limitations in the number of studies, we were not able to specifically explore the possible variability of specific categories of n-3 PUFAs, and further studies are still needed to explore this.

Results from subgroup analysis revealed that TG concentration in serum significantly reduced in studies lasting more than 12 weeks. In our analysis, we concluded that long-term interventions might produce significant lipid-lowering effects. Effects become more pronounced as the duration of the intervention increases. Some specific studies are still needed in the future to explore the possible reasons behind this. Considering that most of the n-3 PUFA supplements used in our current study are purified, and the amount of n-3 PUFAs in different foods is different, we can refer to the intake of different foods according to the recommendations of the Dietary Guidelines for Chinese Residents. For people with different disease states, the dietary guidelines for Chinese residents also give the appropriate food sources and supplemental doses. At present, the best way to supplement n-3 PUFAs is through deep-sea fish rich in n-3 PUFAs, through foods containing fish oils, or by taking supplements to maintain good health.

The present study has some limitations, which we must acknowledge. Firstly, although we searched the relevant literature as much as possible, we still could not avoid some omissions in the literature. Secondly, we considered the effect of n-3 PUFAs in this study and did not consider the effect of different types of interventions due to the relatively small number of included studies. Moreover, we did not explore the dose effect of n-3 PUFAs in depth in the subgroup due to the relatively few studies. Thirdly, the conclusions may be hindered by the risk bias of the trials we included. Finally, we could not perform a test for publication bias due to the limited number of studies.

This meta-analysis also has some advantages. We considered the inclusion of all relevant randomized controlled studies of metabolic syndrome, strictly following the requirements of Cochrane guidelines, with double extraction of data for verification and double execution of literature quality assessment to maximize the quality of the whole study.

## 5. Conclusions

In summary, evidence from our current analysis showed that n-3 PUFA supplementation could decrease serum TG concentration and lower blood pressure among patients with metabolic syndrome. These findings suggest that n-3 PUFA supplementation may serve as a potential dietary supplement for improving lipids and blood pressure in patients with metabolic syndrome. It is still not possible to elucidate in current studies whether there are differences in the effect of specific classes of n-3 PUFAs in improving blood lipids and blood pressure in patients with metabolic syndrome. Additional research should provide further insight.

## Figures and Tables

**Table 1:** The basic characteristics of included trials.

Author	Country	Age, Range (Mean or Median), y	Participants	Study Design	Intervention	Control Group	Intervention Duration	Outcomes
Akrami 2018 [25]	Iran	30–60 (48.6)	52 Participants with metabolic syndrome (26 Experimental group, 26 Control group)	parallel	25 mL flaxseed oil (10.25 mL ALA)	sunflower seed oil	7 weeks	TC, TG, HDL-c, LDL-c, SBP, DBP
Ebrahimi 2009 [26]	Iran	40–70 (42.9)	60 Participants with metabolic syndrome (47 Experimental group, 42 Control group)	parallel	1 g fish oil (180 mg EPA and 120 mg DHA)	blank control	6 months	TC, TG, HDL-c, LDL-c, SBP, DBP
Venturini 2015 [27]	Brazil	NA (51.45)	63 Participants with metabolic syndrome (21 Experimental group, 42 Control group)	parallel	3 g fish oil (1800 mg EPA and 1200 mg DHA)	blank control	90 days	TC, TG, HDL-c, LDL-c
Simao 2014 [28]	Brazil	NA (47.9)	34 women with metabolic syndrome (19 intervention, 15 control)	parallel	3 g fish oil (1200 mg DHA + 1800 mg EPA)	blank control	90 days	TC, TG, HDL-c, LDL-c
Tousoulis 2014 [29]	Greece	NA (44)	29 men with metabolic syndrome	crossover	2 g n-3 PUFAs (46% EPA, 38% DHA)	placebo	12 weeks	TC, TG, HDL-c, LDL-c
Lee 2014 [30]	USA	NA (33.9)	14 metabolic syndromes (7 control, 3 fish oil, 4 Botanical oil)	parallel	9 daily fish oil capsules	corn oil	8 weeks	TC, TG, HDL-c, LDL-c
Tardivo 2015 [31]	Brazil	45–75 (55.1)	63 postmenopausal women with metabolic syndrome (33 intervention, 30 control)	parallel	900 mg n-3 PUFAs (180 mg EPA, 120 mg DHA)	blank control	6 months	TC, TG, SBP, DBP
Xu 2019 [32]	China	18–45 (28.4)	72 metabolic syndromes in patients with schizophrenia (37 intervention, 35 control)	parallel	720 mg EPA and 480 mg DHA	100 mg vitamin E (α-tocopherol)	12 weeks	TG, HDL-c, SBP, DBP

Abbreviations: NA: not applicable; TC: total cholesterol; TG: triglyceride; LDL-c: low-density lipoprotein cholesterol; HDL-c: high-density lipoprotein cholesterol; SBP, systolic blood pressure; DBP, diastolic blood pressure.

## Data Availability

Data is contained within the article and supplementary material.

## Supplementary Materials

The following supporting information can be downloaded at https://www.mdpi.com/article/10.3390/foods12040725/s1: Table S1: Results of Subgroup Analysis; Figure S1: sensitivity analysis of TC; Figure S2: sensitivity analysis of TG; Figure S3: sensitivity analysis of LDL-c; Figure S4: sensitivity analysis of HDL-c; Figure S5: sensitivity analysis of SBP; Figure S6: sensitivity analysis of DBP.

## Appendix A. Detailed Search Strategy

PubMed: (((Cholesterol, HDL[Title/Abstract] OR Cholesterol, LDL[Title/Abstract] OR Triglycerides[Title/Abstract] OR Lipoprotein[Title/Abstract] OR serum lipids[Title/Abstract] OR triglyceride[Title/Abstract] OR low density lipoprotein[Title/Abstract] OR high density lipoprotein[Title/Abstract] OR TG[Title/Abstract] OR TC[Title/Abstract] OR lipid profile[Title/Abstract] OR serum lipid[Title/Abstract] OR oxidative stress[Title/Abstract] OR free radicals[Title/Abstract] OR antioxidants[Title/Abstract] OR blood pressure[Title/Abstract])) AND (fish oils[Title/Abstract] OR omega-3 fatty acids[Title/Abstract] OR polyunsaturated fatty acid[Title/Abstract] OR PUFA[Title/Abstract])) AND ((randomized controlled trial[Title/Abstract] OR randomised controlled trial[Title/Abstract] OR controlled clinical trial[Title/Abstract] OR clinical trial, randomized[Title/Abstract] OR randomised, trial[Title/Abstract] OR randomized[Title/Abstract] OR intervention[Title/Abstract] OR controlled trial[Title/Abstract] OR random[Title/Abstract] OR placebo[Title/Abstract]))

Web of science: TI= (fish oils OR omega-3 fatty acids OR polyunsaturated fatty acid OR PUFA) and AB= (Cholesterol, HDL OR Cholesterol, LDL OR Triglycerides OR Lipoprotein OR serum lipids OR triglyceride OR low density lipoprotein OR high density lipoprotein OR TG OR TC OR lipid profile OR serum lipid OR oxidative stress OR free radicals OR antioxidants OR blood pressure) and AB=(randomized controlled trial OR randomised controlled trial OR controlled clinical trial OR clinical trial, randomized OR randomised, trial OR randomized OR intervention OR controlled trial OR random OR placebo)

Embase: (fish oils OR omega-3 fatty acids OR polyunsaturated fatty acid OR PUFA) and (Cholesterol, HDL OR Cholesterol, LDL OR Triglycerides OR Lipoprotein OR serum lipids OR triglyceride OR low density lipoprotein OR high density lipoprotein OR TG OR TC OR lipid profile OR serum lipid OR oxidative stress OR free radicals OR antioxidants OR blood pressure) and (randomized controlled trial OR randomised controlled trial OR controlled clinical trial OR clinical trial, randomized OR randomised, trial OR randomized OR intervention OR controlled trial OR random OR placebo)

Cochrane library: (fish oils OR omega-3 fatty acids OR polyunsaturated fatty acid OR PUFA) and (Cholesterol, HDL OR Cholesterol, LDL OR Triglycerides OR Lipoprotein OR serum lipids OR triglyceride OR low density lipoprotein OR high density lipoprotein OR TG OR TC OR lipid profile OR serum lipid OR oxidative stress OR free radicals OR antioxidants OR blood pressure) and (randomized controlled trial OR randomised controlled trial OR controlled clinical trial OR clinical trial, randomized OR randomised, trial OR randomized OR intervention OR controlled trial OR random OR placebo)
