# Supplementary material for: Effects of Omega-3 Fatty Acids Supplementation on Serum Lipid Profile and Blood Pressure in Patients with Metabolic Syndrome: A Systematic Review and Meta-Analysis of Randomized Controlled Trials
Source: Foods. 2023 Feb 7;12(4):725. doi: 10.3390/foods12040725 (PMC9956263; doi:10.3390/foods12040725)
Supplement: Supplementary file 1 [file foods-12-00725-s001.zip › foods-2145935-supplementary.pdf]

Supplementary Materials

Supplement to:

Effects of omega-3 fatty acids supplementation on serum lipid profile and blood pressure in patients with metabolic syndrome: a systematic review and meta-analysis of randomized controlled trials

Yin-xiu Liu, Jun-hui Yu, Ji-han Sun, Wen-qin Ma, Jin-jing Wang, Gui-ju Sun

Table S1. Results of Subgroup Analysis

Index	Subgroup	No. of Trials	Standardized mean difference		*P*	I2(%)	*P* value of heterogeneity
			mean	*95%CI*			
**TC**	Overall	7	-0.02	-0.22, 0.18	0.86	23.7	0.248
	Intervention duration						
	＜12 weeks	2	0.38	-0.13, 0.90	0.141	14.6	0.279
	＞=12 weeks	5	-0.09	-0.31, 0.13	0.411	0	0.421
	Control type						
	oil or placebo	3	0.01	-0.35, 0.38	0.946	62.2	0.071
	Blank control	4	-0.03	-0.27, 0.21	0.798	0	0.468
	Study design						
	parallel	6	0.04	-0.17, 0.26	0.692	13.6	0.327
	crossover	1	-0.37	-0.89, 0.15	0.162	0	-
	Study quality						
	Low risk	3	-0.18	-0.50, 0.14	0.26	45.2	0.161
	High or unclear risk	4	0.09	-0.17, 0.35	0.492	0	0.474
**TG**	Overall	7	-0.39	-0.59, -0.18	＜0.001	17.2	0.298
	Intervention duration						

	< 12 weeks	2	-0.08	-0.59, 0.42	0.748	0	0.339
	＞=12 weeks	5	-0.45	-0.67, -0.22	< 0.001	14.8	0.32
	Control type						
	oil or placebo	4	-0.45	-0.74, -0.17	0.002	26.5	0.253
	Blank control	3	-0.31	-0.61, -0.01	0.042	25.7	0.26
	Study design						
	parallel	6	-0.33	-0.55, -0.11	0.004	10.5	0.348
	crossover	1	-0.71	-1.24, -0.18	0.009	100	-
	Study quality						
	Low risk	4	-0.56	-0.82, -0.30	< 0.001	0	0.893
	High or unclear risk	3	-0.11	-0.44, 0.22	0.527	9.2	0.332
**LDL-c**	Overall	5	0.18	-0.18, 0.53	0.325	54.9	0.065
	Intervention duration						
	< 12 weeks	1	0.35	-0.20, 0.90	0.210	0	-
	＞=12 weeks	4	0.14	-0.30, 0.58	0.525	63.6	0.041
	Control type						

	oil or placebo	2	-0.05	-0.82, 0.72	0.901	76.1	0.041
	Blank control	3	0.31	-0.04, 0.65	0.086	23.9	0.269
	Study design						
	parallel	4	0.3	0.04, 0.56	0.024	0	0.445
	crossover	1	-0.44	-0.96, 0.08	0.099	0	-
	Study quality						
	Low risk	1	-0.44	-0.96, 0.08	0.099	0	-
	High or unclear risk	4	0.3	0.04, 0.56	0.0224	0	0.445
**HDL-c**	Overall	5	0.02	-0.21, 0.25	0.882	0	0.645
	Intervention duration						
	＜12 weeks	1	-0.09	-0.64, 0.45	0.734	0	-
	＞=12 weeks	4	0.04	-0.21, 0.30	0.746	0	0.522
	Control type						
	oil or placebo	2	-0.04	-0.42, 0.33	0.815	0	0.805
	Blank control	3	0.06	-0.24, 0.35	0.709	9.8	0.33
	Study design						
	parallel	4	0.02	-0.24, 0.28	0.868	0	0.486
	crossover	1	0	-0.51, 0.51	1	0	-

	Study quality						
	Low risk	1	0	-0.51,0.51	1	0	-
	High or unclear risk	4	0.02	-0.24, 0.28	0.868	0	0.486

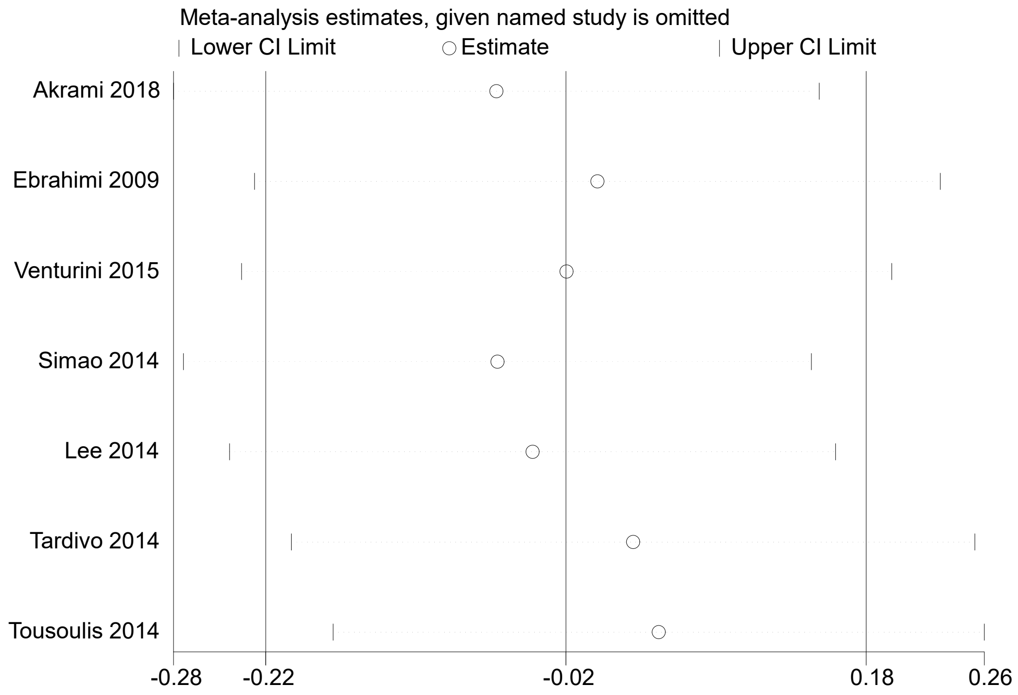

Figure S1. sensitivity analysis of TC

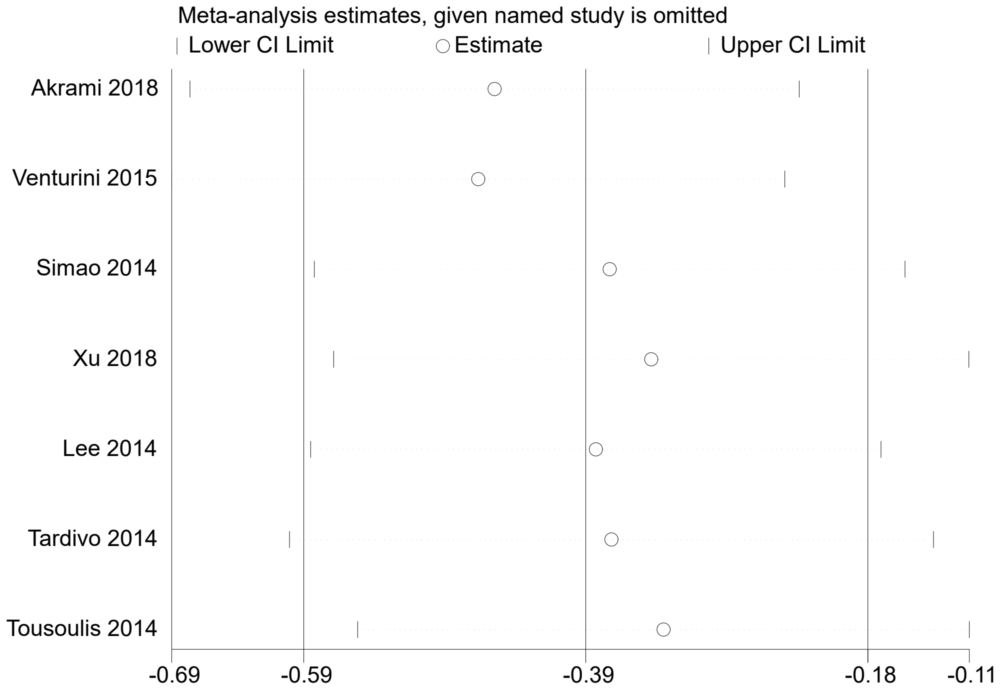

Figure S2. sensitivity analysis of TG

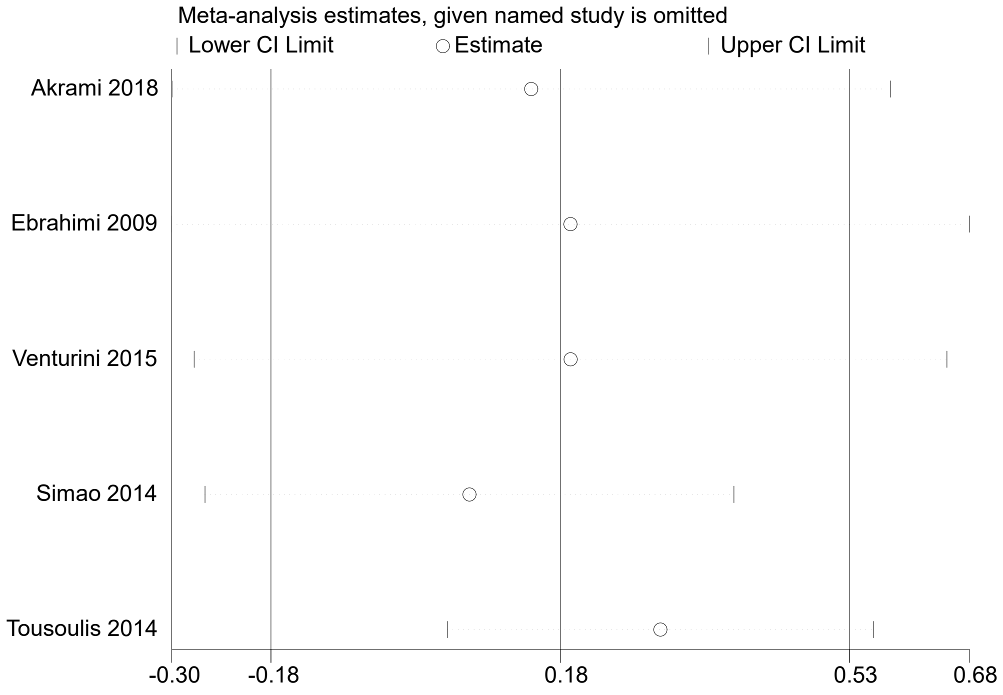

Figure S3. sensitivity analysis of LDL-c

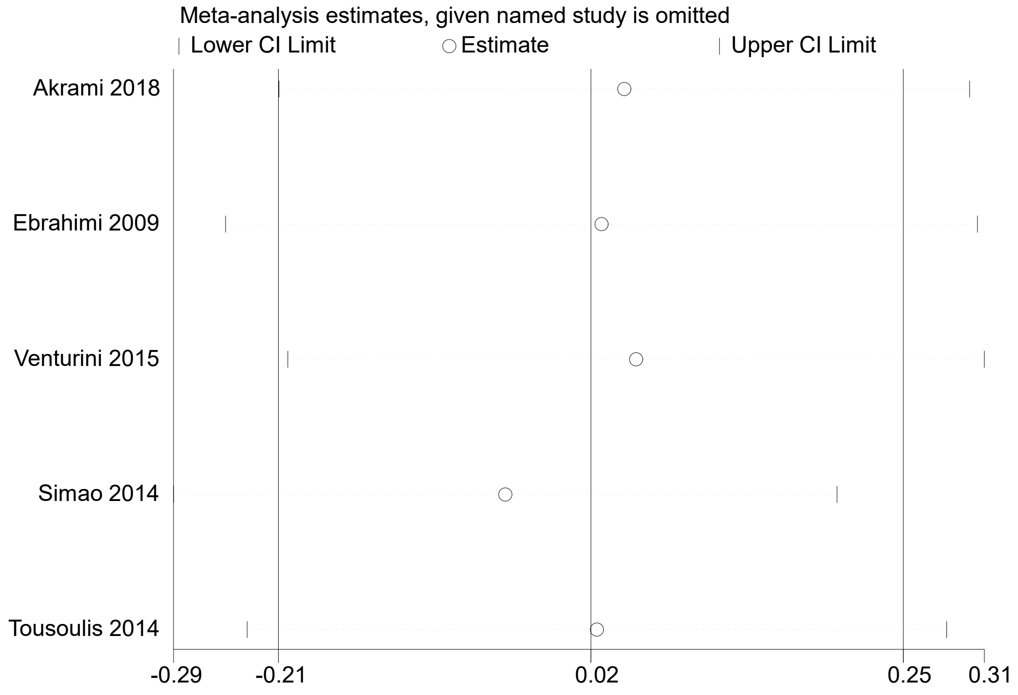

Figure S4. sensitivity analysis of HDL-c

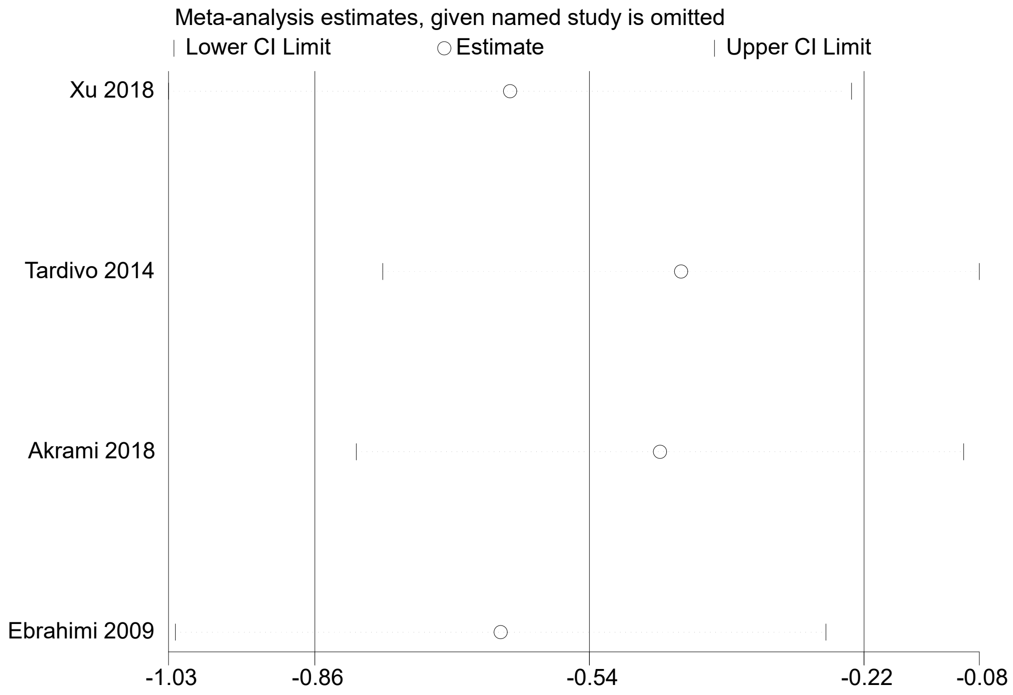

Figure S5. sensitivity analysis of SBP

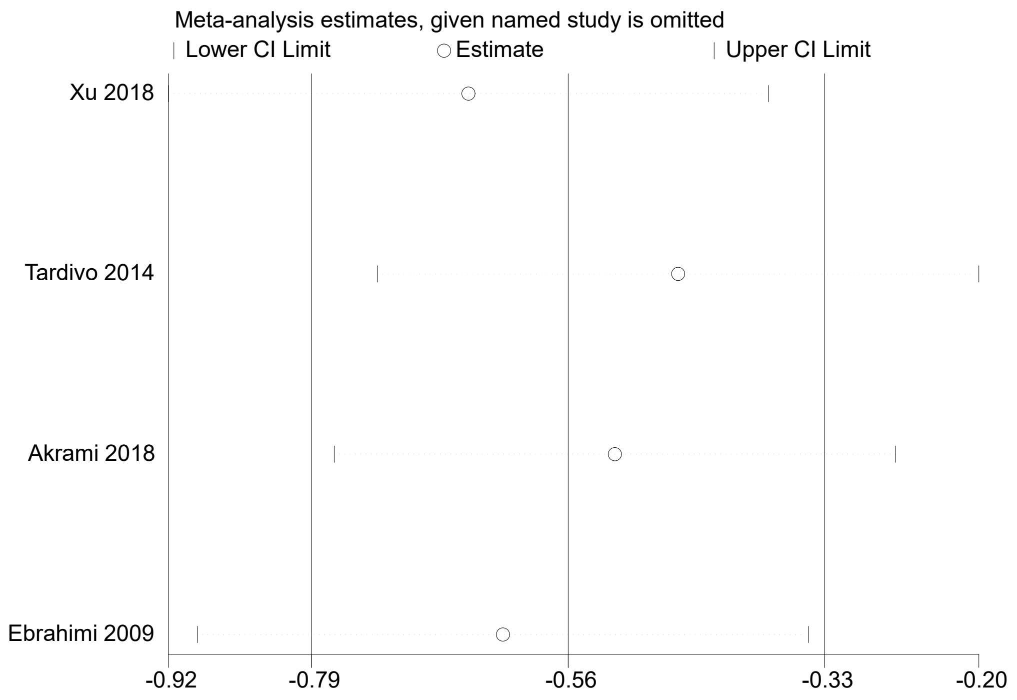

Figure S6. sensitivity analysis of DBP
